# Dynamics of Potassium Release and Adsorption on Rice Straw Residue

**DOI:** 10.1371/journal.pone.0090440

**Published:** 2014-02-28

**Authors:** Jifu Li, Jianwei Lu, Xiaokun Li, Tao Ren, Rihuan Cong, Li Zhou

**Affiliations:** 1 College of Resources and Environment, Huazhong Agricultural University, Wuhan, China; 2 Key Laboratory of Arable Land Conservation (Middle and Lower Reaches of Yangtse River), Ministry of Agriculture, Wuhan, China; German Cancer Research Center, Germany

## Abstract

Straw application can not only increase crop yields, improve soil structure and enrich soil fertility, but can also enhance water and nutrient retention. The aim of this study was to ascertain the relationships between straw decomposition and the release-adsorption processes of K^+^. This study increases the understanding of the roles played by agricultural crop residues in the soil environment, informs more effective straw recycling and provides a method for reducing potassium loss. The influence of straw decomposition on the K^+^ release rate in paddy soil under flooded condition was studied using incubation experiments, which indicated the decomposition process of rice straw could be divided into two main stages: (a) a rapid decomposition stage from 0 to 60 d and (b) a slow decomposition stage from 60 to 110 d. However, the characteristics of the straw potassium release were different from those of the overall straw decomposition, as 90% of total K was released by the third day of the study. The batches of the K sorption experiments showed that crop residues could adsorb K^+^ from the ambient environment, which was subject to decomposition periods and extra K^+^ concentration. In addition, a number of materials or binding sites were observed on straw residues using IR analysis, indicating possible coupling sites for K^+^ ions. The aqueous solution experiments indicated that raw straw could absorb water at 3.88 g g^−1^, and this rate rose to its maximum 15 d after incubation. All of the experiments demonstrated that crop residues could absorb large amount of aqueous solution to preserve K^+^ indirectly during the initial decomposition period. These crop residues could also directly adsorb K^+^ via physical and chemical adsorption in the later period, allowing part of this K^+^ to be absorbed by plants for the next growing season.

## Introduction

The current Asian population of 4.3 billion is projected to increase by nearly 0.9 billion people, reaching roughly 5.2 billion, by 2050 [1], which will result in significantly increased regional food demand. Of this, population, 80% will be distributed in China, India and the southeast regions of Asia, posing a challenge to the economic development and social stability of these countries [2]. Rice-based cropping systems are the most productive agroecosystems in these areas and produce the most food for the most people [3]. To meet the food demand of the region, intensification and diversification have been applied as the two main strategies for rice-based cropping systems. In addition to the rise of multiple cropping indexes, fertilization consumption has played a very important role in production increases [4]. Compared with nitrogen and phosphorus fertilizer, potash fertilizer is often ignored by farmers, particularly in Asia [5]–[7]. Potash resources are comparatively limited [8], [9], and in recent years, the higher price of potash on the international market has reduced the demand of potassium, as farmers in the area are unwilling to put more potash into the soil [10]. Soil K deficiency has become a major limiting factor in the modern agricultural process [11]. Therefore, it is of great importance to increase potash supplementation in these regions. K-bearing organic resources such as compost, green manure, farmyard manure and crop straws, particularly abundant crop residues, are again receiving attention from farmers [12].

Annually, the world production of straw is approximately 3.8 billion tons, 74% of which are cereal straws [13]; for rice-based land in Asia, 80% of straw production consists of rice residues [3]. Cereal straws usually have a higher potassium content than other straws (1.2%–1.7%). The results of Kaur and Benipal [14] have shown that returning straw to the field could improve soil available potassium to a significantly great extent than manure. A 30-years field trial conducted by Liao et al. indicated that straw management could increase exchangeable K by 26.4%, nonexchangeable K by 1.8% and SOC 21.0% in comparison to a CK treatment in reddish paddy soil [15]. As straw potassium is primarily present in the form of K^+^ ions in the cell fluid [16], the release of K from stubble in field is influenced by rainfall [17]. Duong et al. [18] found that the distance of potassium migration from organic fertilizer is 10 mm. Excepting the K^+^ adsorbed or fixed by soil clay particles, 50% of K^+^ was retained in the soil solution [14]. Furthermore, farmers prefer to input potash fertilizers one time before sowing. The loss of K in the soil solution from such applications was 1.1- and 14.5- fold that of N and P, respectively [19]; N and P losses were gradual, while leaching phenomena were observed for K [17]. However, Kozak et al. found that crop residues could intercept a maximum of 29% of the water loss for a given rainfall [20]. Soil water retention is also affected by the organic carbon content, as reported by Rawls et al [21]. These results indicate that crop residues have a positive effect on water absorption. Meanwhile, as a high-quality biological adsorbent, the biochars generated from crop straws can adsorb 0.48–1.40 mol kg^−1^ Cu(II) [22], and even unmodified rice straw can absorb 13.9 mg g^−1^ Cd(II) [23]. However, relatively few data exist regarding the adsorption of potassium by crop residues. Because straw decomposition is a slow and long-term process, a significant quality of plant residues can usually be found on farmland after one season of crop growth.

Since 1980, global warming has received increasing attention [24], bolstered by occasional extreme weather events such as the sustained hot temperature in Europe, North America and Asia in July 2013. Scorching weather causes a water shortage in rice farmland, thereby affecting the absorption of nutrients. The incorporation of residues into the soil may reserve water to slow down seasonal drought and could also adsorb cations. However, the capability of soil residues to preserve water and nutrients during different decomposition periods is unclear, especially, the fixation mechanism of residues for potassium.

Therefore, the aims of this research were as follow: (a) to investigate the characteristics of straw decomposition and K^+^ release under flooded conditions; (b) to assess the retention capacity of straw for water and potassium during different decomposition periods; and (c) to ascertain the mechanism of K^+^ adsorption on straw residues.

## Materials and Methods

### Ethics Statement

The authors of this study hereby confirm that no specific permissions were required for our experimental location and activities, as the experimental field belonged to our institute and is employed for scientific research only. The studies had negligible effects on the functioning of the broader ecosystem. The research did not involve measurements on humans or animals, and no endangered or protected species were involved.

### Materials

#### Soil material

Paddy soil was collected from the plow layer (0–20 cm) after the rapeseed harvest in 2012. The sample was mixed, air-dried at room temperature and ground to pass through a 2 mm sieve. Soil physical and chemical properties were measured using conventional methods [25]–[27]. Soil pH was measured with a glass electrode in a 1∶2.5 soil/water solution. Soil organic matter was measured using the dichromate oxidation method, and total nitrogen was measured using the Kjeldahl acid digestion method. Available phosphorus was determined using the Olsen method, and available potassium was measured by flame photometry after NH_4_OAc neutral extraction. All parameters were measured three times and are presented as the mean±SD: pH 5.73±0.23, SOM 26.7±1.1 g kg^−1^, TN 1.09±0.04 g kg^−1^, Olsen-P 19.4±0.7 mg kg^−1^, and NH_4_OAc-K 107.8±4.3 mg kg^−1^.

#### Rice straw material

Rice straw was cut into segments of approximately 2–3 cm and then conserved in a dryer until further use. The initial potassium (K) content of the rice straw was 21.87±0.12 mg g^−1^.

### Methods

#### Straw decomposition trial

The trial was performed at the experimental base of the College of Resources and Environment beginning on July 7, 2012. Three boxes, constructed of PVC (size 50×30×25 cm), were used for the incubation experiment. A total of 15 kg of dry bulk soil was packed in each box. Before the experiment, the rice straw was dried at 40°C for 3 h, and 10.0 g samples were then accurately weighed into 200 mesh (pore diameter 0.075 mm) nylon bag (size 25×20 cm), and sealed [28]. Each box contained 5 bags of straw, totaling 15 bags in the three boxes. According to the growth period of late rice, we removed nylon bags at 5, 15, 30, 60 and 110 d. During the incubation period, deionized water was added to the boxes to maintain a flooding layer of 1 cm. A schematic diagram of the trial is shown in Figure S1.

On each sampling date, one nylon bag was randomly removed from each box and rinsed with distilled water three times to remove any mud that had adhered to the bag. The residue of the rice straw was then dried at 40°C for 48 h until reaching a constant weight, weighed and ground to pass through a 1 mm sieve. Some of the residue powder was ground again using a mortar, until it passed through a 0.149 mm sieve, to obtain micron particles. The particles were dispersed in an aqueous solution of pH 6.0 to test the zeta potential using zeta potential and nanoparticle size analyzer (ZS90, Melvin British Company UK). Zeta potential is the potential difference between the dispersion medium and the stationary layer of fluid attached to the dispersed particle [29], which indicates the electric potential variation of the residue surface during the decomposition period [22]. All of the residue samples were digested with H_2_SO_4_-H_2_O_2_ to determine their potassium content using a flame photometer (M-410, Cole-Parmer USA) and to calculate their potassium release rates. The formulas employed in this study were as follow:



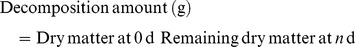
(1)




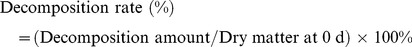
(2)

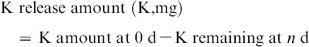
(3)




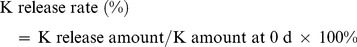
(4)Where *n* is the day of incubation.

#### Batches of K sorption experiments

Precise 0.30 g samples of the rice straw residue from different time points were added into 50 mL polythene bottles along with various concentrations of KCl solution (0, 10, 50 and 100 mg K L^−1^). The bottles were shaken for 4 h at 160 r min^−1^ and filtered to test the K^+^ concentration of the equilibrium solution. The experiments were repeated 3 times, and the average values were used for analysis. The K adsorption on straw Q (mg g^−1^) and the K removing rate R (%) were determined in the following manner [30]:
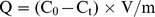
(5)




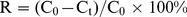
(6)Where C_0_ and C_t_ are the initial and equilibrium K concentration, respectively, mg L^−1^; V is the volume of the solution, mL; and m is the mass of the straw residue, g.

According to the K sorption experiment, the maximal Q residue was selected to test the changes of residue structure on K^+^ fixation with the help of Fourier transform infrared spectroscopy. (Nexus, Thermo Nicolet USA).

#### Kinetics of water absorption on rice straw

The water absorption capacity of untreated dried straw [31] was determined by suspending 0.50 g of natural dried straw through a 1.0 mm sieve in a 50 mL beaker with 40 mL deionized water. three replicates were taken at each time interval and cleaned of gravitational water from the straw surface. The material was then weighed on an electronic balance, and the water absorption capacity was calculated.

The water absorption capacity of rice straw residues from different decomposition time points was also determined. A straw sample of 0.30 g was put into a 50 mL beaker with 40 mL of deionized water. After 300 min of immersion, the residues were removed and cleaned of gravitational water before the determination of water absorption capacity. The water absorption capacity of the straw was calculated as follows:
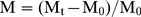
(7)Where M is the water absorption capacity of straw, g g−1; and Mt and M0 are the initial and final mass of straw, respectively, g.

Furthermore, to observe the influence of straw surface pore on the water absorption of rice straw, the surface morphology of residue from different time points was examined using a scanning electron microscope (JSM-6390LV, NTC Japan).

### Data Analysis

All analyses were conducted on three replicates. Statistical analyses were performed using OrignPro8.0 software. The means were the average of three replicates, and analysis of variance (ANOVA) was performed according to standard procedures for factorial randomized block designs. Differences at p<0.05 level were considered statistically significant, as determined using the least significant difference (LSD) test.

## Results

### Characteristics of Straw Decomposition and Zeta Potential of Rice Straw Surface

**Figure 1:**
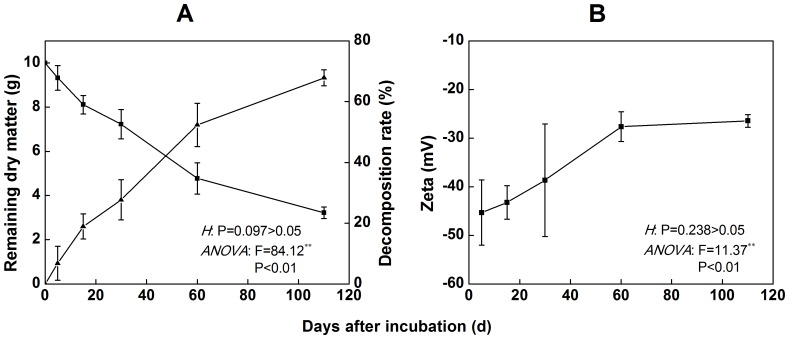
The characteristics of the decomposition and zeta potential of rice straw. The annotations in the panels indicate the homogeneity of variances (H) and ANOVA for the remaining dry matter and zeta potential. The H-test was performed using the Levene test. **indicates significant differences at P<0.01. The values are the means of 3 replicates (±standard deviation).

The decomposition dynamics of rice straw during different stages of incubation under flooded conditions are shown in Figure 1A. The average rate of rice straw decomposition was 0.09 g d^−1^ from 0 to 60 d. The decomposition amount accounted for 52.3% (remaining mass 4.77 g) of the total mass after 60 d of incubation. The average rate of decomposition was relatively slow, 0.03 g d^−1^, from 60 to 110 d. During this period, the decomposition amount accounted for 15.5% of the total mass. The remaining amounts of straw and the accumulation rate were 3.22 g and 67.8%, respectively, after the trial was ended at 110 d.

The zeta potential of the untreated rice straw surface was −56.36 mV in the pH 6.0 aqueous phase (Figure 1B). After 5 d of incubation, the zeta potential increased to −43.2 mV, and it continued to increase to −27.65 mV after 60 d of incubation. On the following days, the zeta potential was not significantly different, ranging from −30 to −20 mV.

### Characteristics of Straw K Release

**Figure 2:**
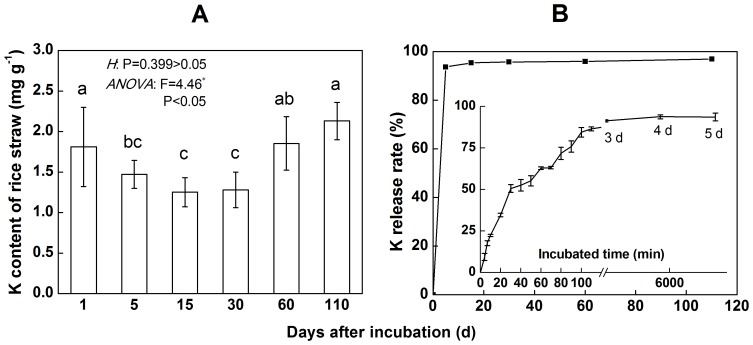
The characteristics of the potassium release of rice straw. The annotation in panel A indicates the homogeneity of variances (H) and ANOVA. The H-test was performed using the Levene test. *indicates significant differences at P<0.05. The values are the means of 3 replicates (±standard deviation). Means with the same letter are not significantly different. The insertion in panel B is the K release rate within 5 d.

The potassium (K) content of untreated rice straw was 21.87 mg kg^−1^. After 3 d of immersion, 90% of the total K had been released, and the K content of straw was only 1.47 mg g^−1^ after 5 d of immersion (Figure 2A); this value did not change significantly on the following days. These results indicated that 93.7% of the total potassium could be released into the ambient environment during the early stage of immersion (Figure 2B). After 30 d, the K content of the residues increased slightly and remained at 1–2 mg g^−1^. At the end of the trial, the K content of the residue and the K release rate were 2.13 mg g^−1^ and 96.9%, respectively.

### K Adsorption in Different Decomposition Periods of Straw Residue

**Figure 3:**
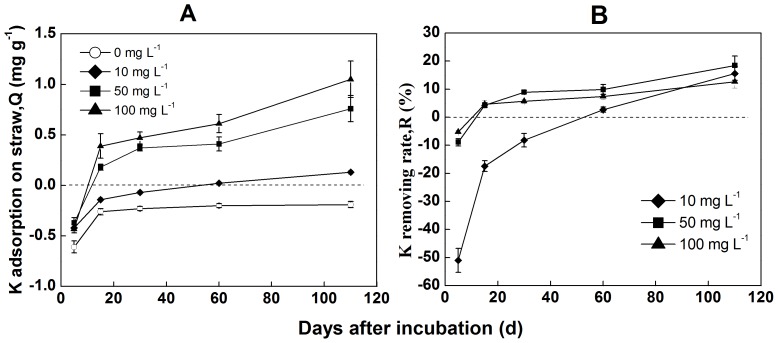
The adsorption of potassium by rice straw residues. The dashed line shows the zero point in the panels. The values are the means of 3 replicates (±standard deviation).

Figure 3 shows the adsorption-desorption equilibrium of exogenous potassium over different decomposition periods of straw residue. The results showed that over the entire decomposition period, K^+^ ions were, on the whole, released in pure water. When the concentration of K^+^ ions was 10 mg L^−1^ in solution, a positive effect of K^+^ adsorption was observed after 60 d of decomposition, and the K^+^ adsorption capacity of the residue reached 0.13 mg g^−1^ on the 110^th^ d of the experiment. The residue at 15 d showed a positive adsorption effect when the concentration of external potassium increased to 50 mg L^−1^. Meanwhile, the capacity also increased as decomposition continued. The amount of K^+^ adsorption reached a maximum of 0.76 mg g^−1^ after 110 d of incubation, representing a considerable increase of 76.3% compared to its value on the 15^th^ day. When additional increments of K were applied, up to 100 mg L^−1^, the straw primarily released potassium into solution within 5 d. After 15 d, the adsorption capacity of the residue was higher than that in the trial with 50 mg L^−1^ K supplied over the same period.

Similarly, results of Figure 3B indicated that the K removing rate (R) increased with the decomposition of rice straw. When the concentration of extra K was 10 mg L^−1^, for example, the value of R was −51.0% at 5 d after incubation, but reached 15.6% after 110 d. At the same time, when the concentration of external K was increased to 50 mg L^−1^, the removing rate for each period of decomposition except for the first 5 d reached the maximum value. The values of R tended to decrease with increase external K concentration.

### FT-IR Analysis for the K Adsorption on Rice Straw Residue

**Figure 4:**
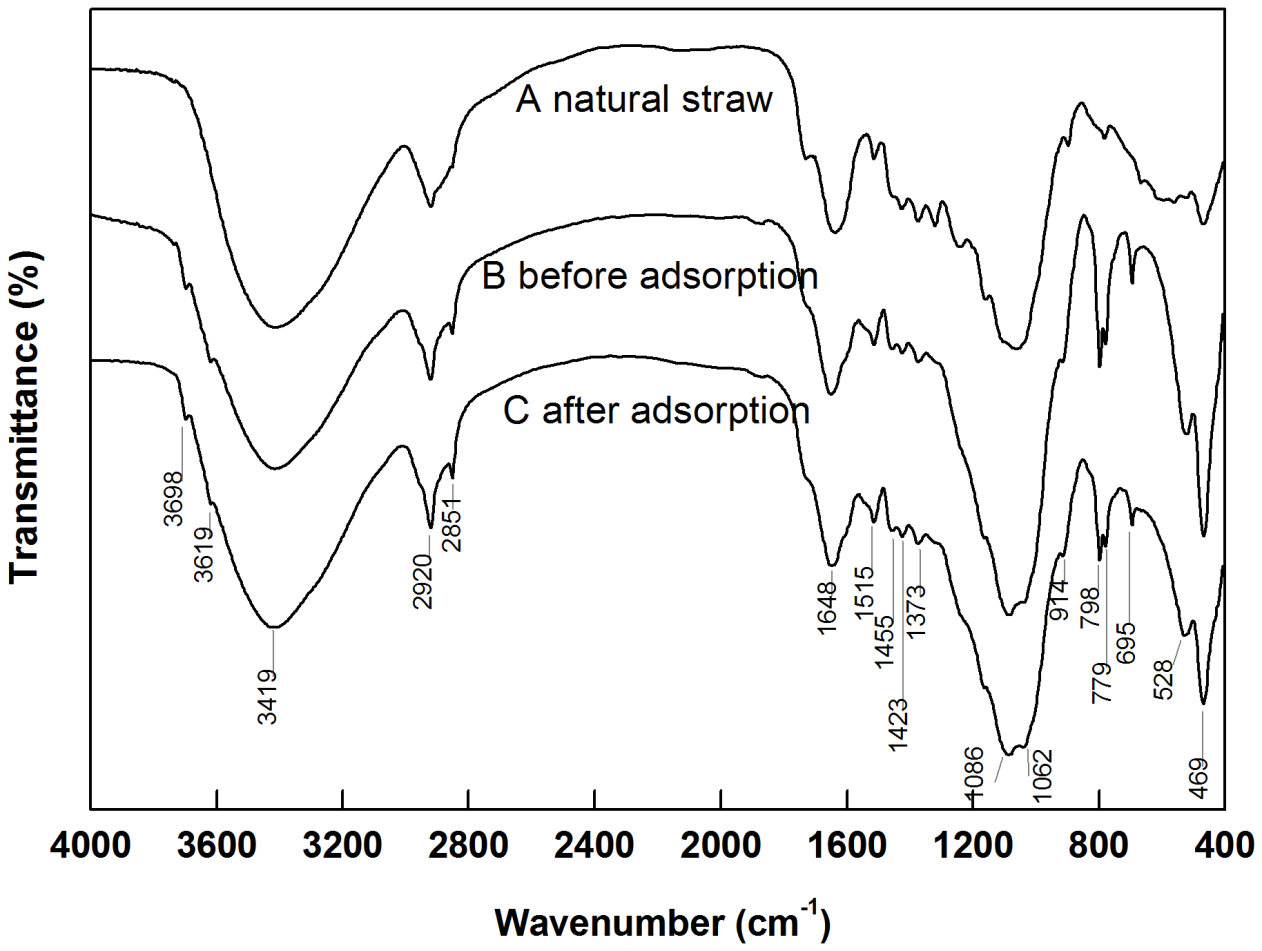
FT-IR spectra of natural dried rice straw and the rice straw residue at 110 d before and after potassium (K) adsorption. Solid line A represents the infrared spectrum of natural dried rice straw. Solid lines B and C represent the infrared spectra of the rice straw at 110^−1^ KCl solution, respectively. The data in the panel are the peak wavenumbers.

Figure 4 displays the infrared spectra of untreated dried straw both before and after the K adsorption of the rice straw residue on the 110^th^ day after incubation. The results showed that the chemical structure of the straw had undergone a significant change after 110 d of decomposition (Figure 4A–B). New peaks emerged at 3698, 3619 and 779 cm^−1^, and the existing peaks at 2851, 1086, 798, 695, 528 and 469 cm^−1^ were strengthened to various degrees.

Figure 4C illustrates the spectra after K adsorption for rice straw after 110 d. The main peaks of the functional groups at 4000−1300 cm^−1^ did not show significant changes. However, in the fingerprint region, the peaks at 798, 695, and 469 cm^−1^ decreased significantly compared to those before K adsorption on the residue.

### Kinetics of Water Absorption on Rice Straw

**Figure 5:**
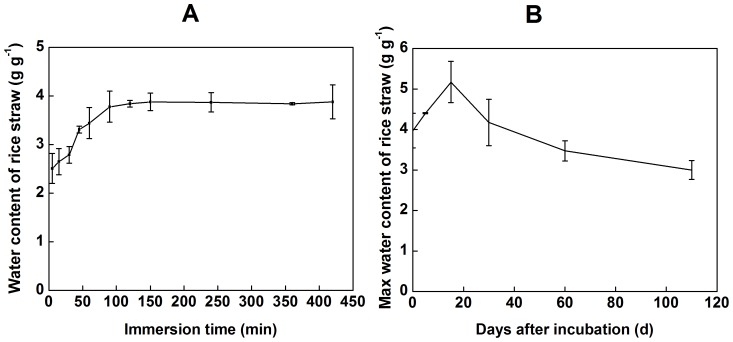
The kinetics of water absorption for rice straw. Panel A shows the changes of water absorption in dried rice straw. Panel B shows the changes of water absorption of straw residue for different decomposition periods. The values are the means of 3 replicates (±standard deviation).

The water absorption kinetic of untreated dried straw are shown in Figure 5A. The results indicated that the water storage capacity of straw reached 2.51 g g^−1^ after 5 min of immersions. From 5 to 90 min, the absorption rate continued to increase at an average rate of 0.01 g (g min) ^−1^. After 150 min, the rice straw could not absorb more water at the ambient temperature (23°C), approaching a saturated value of 3.88 g g^−1^. The water retention also changed significantly during different periods of straw decomposition (Figure 5B). The results showed that the water absorption capacity of the residue continued to rise from 0 to 15 d, and the maximum water content was 5.17 g g^−1^. Moreover, the water absorption capacity decreased gradually with the extension of straw decomposition. The water content of rice residue was the lowest at 110 d, with a value of 3.00 g g^−1^.

## Discussions

### The Differences between Straw Decomposition and K Release

**Figure 6:**
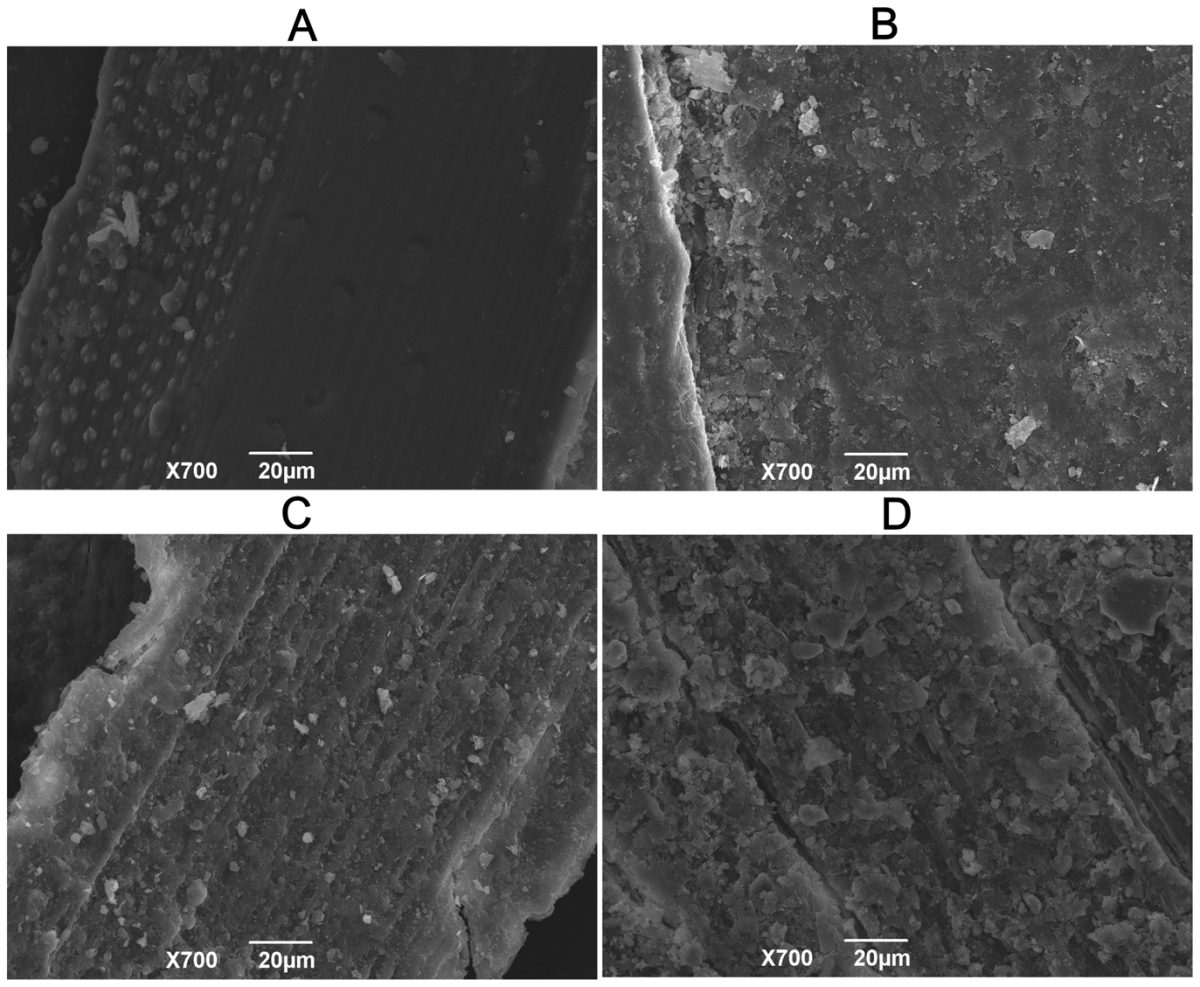
SEM micrographs of the rice straw residue surface on different days after incubation: A 0 d, B 15 d, C 60 d and D 110 d.

For field straw management, the rate of straw decomposition is influenced by many factors. These variables include internal factors, such as litter quality, material composition and structure [32], [33], and external factors, such as temperature, moisture [34], methods of straw utilization (mulching or incorporation), time, length of straw [31] and the concentration of CO_2_ in the soil. Litter quality and climate condition are considered to be key factors in the regulation of straw decomposition [35]. The present study was conducted from the beginning of July to the end of October. From 0 to 60 d, the soil temperature reached above 35°C, and this environment did significantly improve the decomposition rate of the straw. However, from 60 to 110 d, the soil temperature decreased to 20–24°C, and the decomposition rate consequently also decreased. After 110 d of degradation, the straw decomposition rate reached 60%. The results presented in Figure 1 illustrate that the decomposition of straw has two main periods: (1) a rapid decomposition stage from 0 to 60 d, during which the structure of the straw surface changed (Figure 6), and the zeta potential increased significantly; and (2) a slow decomposition stage from 60 to 110 d, during which the decomposition rate is significantly lower than before and the zeta potential is basically stable. The characteristic infrared peaks of cellulose were observed at 3412, 2900, 1425, 1370 and 895 cm^−1^ [30]. The characteristic peak of hemicellulose was observed at 1732 cm^−1^ [36], and the absorption peaks characteristic of lignin were seen at 1595 and 1516 cm^−1^. All of these peaks were not changed dramatically over the study period, as seen in figure 4. Conversely, easily decomposable plant materials such as lipids, pectin, starch and carbohydrates were degraded over one season of crop growth. In addition, negligible amounts of cellulose and hemicellulose were also degraded. Lignin, along with the majority of the cellulose and hemicellulose, may require more time to be degraded by organisms [35].

The characteristics of K release are dramatically different from those of straw decomposition. As K mainly exists in ionic form in plants, it is able to move easily. After 5 d of immersion, more than 90% of potassium had been released from the straw. This result coincides with that of Rodríguez-Lizana et al. [17], who reported that the K of sunflower residue decreased by 98% over the study period while only 43% of the residue itself degraded over the same time. Thus, the maintenance of residues under conservation tillage requires the application of large amount of K to the soil.

### Mechanism of K Adsorption on Straw Residues

Currently, the ability of crop straw to serve as a bioadsorbent material has been widely studied and has received increasing attention, especially regarding the possibility of reducing the pollution of heavy metals such as Hg(II), Cd(II), and U(VI) [23], [37]. The adsorption of metal ions mainly relies on physical adsorption and chemical adsorption. Usually, the latter process is dominated, as reported by Ke et al., who observed that modified rice straw coupled with metal ions to form organic compounds [38]. However, few report exist regarding the coupling interaction between heavy metals and residues during different decomposition period. In particular, alkali metal atoms (Li, Na, K) rarely form complexes with organic ligands because of their unique structures (which lack an empty orbital in the valence shell). When the active functional groups in rice straw bound Cd, nearly all the infrared bands from 3423 to 1000 cm^−1^ decreased in intensity [23]. In the residues after K adsorption, the functional group signatures at 4000 to 1300 cm^−1^ did not change (Figure 4). These results indicate that potassium chelated with C = C, C-O or carboxylic acids on straw is impossible. Moreover, greater focus has been placed in previous studies on the changes in the physical or chemical properties of soil after straw management [39], or on the conversion among diverse forms of nutrients [11]; the straw residue itself has therefore often been ignored. The adequate prediction of K dynamics and bioavailability is further complicated by the limited knowledge of the reversibility of K adsorption in soil. However, it was noted in the present study that rice straw residue could adsorb potassium despite releasing most of its potassium at the beginning of the experiment. At the same time, rape (canola) and wheat straw at different points in decomposition could also adsorb a portion of the K^+^ ions, indicating that the role of crop residues in potassium adsorption is widespread (Figure S2). The present study provides both direct and indirect observations demonstrating that the adsorption mechanism can be described by the following three aspects:

1. Physically interfacial adsorption: electrostatic adsorption. Due to higher content of potassium in natural straw, the rate of K release is significantly greater than the rate of K adsorption capacity during the earlier stage of decomposition. As decomposition continues, the zeta potential of the straw surface increases. Straw residues could adsorb K^+^ ions from the surrounding environment (Figure 3). The straw K adsorption capacity is a function of the decomposition period and the extra K concentration, indicating an equilibrium between the K pool adsorbed on the residue and that in the bulk solution. Residues had an especially strong adsorption capacity after long-term decomposition. Therefore, as vast majority of K fertilizer inputs to the soil are one-time events, a portion of these inputs could be replaced by accumulated debris (or organic matter). Considering the K uptake by plants when the K concentration in the soil solution is reduced, crop residues could release K to replenish the soil solution [18].
2. Chemically interfacial adsorption. The infrared spectra of straw residues after K adsorption on the 110^th^ day of decomposition showed decreased intensity of the peaks at 798, 695 and 468 cm^−1^ in the fingerprint region. This results suggested that binding sites existed on the residue surface that could couple with K^+^ ions. For peak at 798 cm^−1^ indicates the presence of C-H stretching or Si-O stretching, and Lu et al has demonstrated that the peaks at 795 and 466 cm^−1^ represent symmetric Si-O stretching vibration and Si-O deformation vibration, respectively [40]. The peak at 695 cm^−1^ likely reflects the vibration of nitrogenous or boric compounds [41], [42]. These results confirmed that the compounds containing Si, B and N on the residue surface were involved in coupling with K^+^.
3. Solution fixed on residue. A study of the water retention of the 11 main varieties of crop straw, as conducted by Iqbal et al., showed that the water retention of straw was not necessarily linked with content of its components (cellulose, hemicellulose, lignin) but was significant related with its porosity and the variation of this porosity during decomposition. These authors found that the water content of rice straw reached its maximum after 6 h of immersion [31]. According to the present research, the capacity of water absorbed on the straw reached saturation after 150 min of immersion, which was consistent with the pervious study. The water content of the decomposed residue reached its maximum of 5.17 g g^−1^ after 15 d of incubation. Due to the differences of porosity, the change of the water content of the decomposed rice residue differed from that of maize residue, which showed higher water retention as incubation proceeded [31]. On the fifth day, the rice straw displayed a regular and compact surface structure, showing no difference with the raw material [43], which likely relied on its inner surface to absorb water (Figure 6). After 15 d of incubation, pectin, starch and other biodegradable materials on the surface of straw were depredated and fell off; the resulting surface was rougher and retained more water. After 60 d, the easily decomposable materials on the straw surface were completely decomposed, and the lignin and cellulose were exposed to the surface. Due to the reduction of porosity, the water retention decreased.

**Figure 7:**
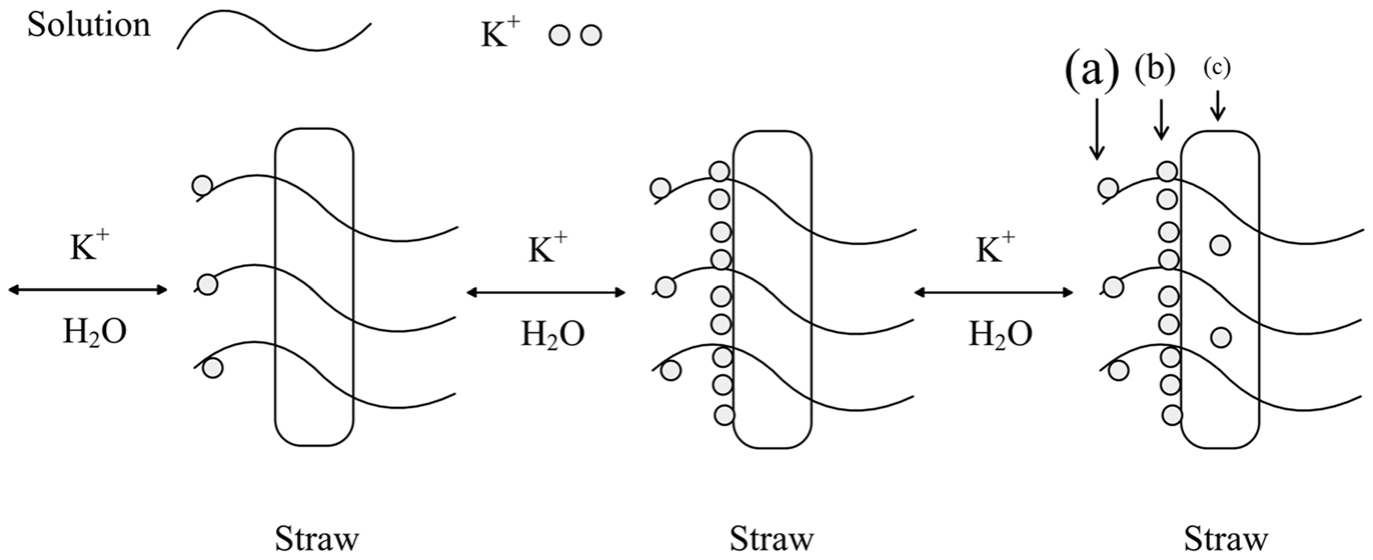
Schematic of the adsorption process of potassium (K^+^) on the rice straw residue surface. (a) K^+^ retained on the straw in the form of an aqueous solution absorbed by residue; (b) K^+^ adsorbed via electrostatic interaction; and (c) K^+^ fixed on the residue surface by chemically interfacial adsorption. The size of the three letters indicates the amount of K^+^ reserved on the residue by the corresponding process.

Briefly, the K adsorption process can be described with the following steps (Figure 7): (1) K^+^ is transferred from the solution to the rice straw surface; (2) fractional K^+^ ions are transferred from the rice straw surface to active sites; and (3) the water content on the surface is maximized, and the K^+^ on the residue surface reaches equilibrium with the bulk solution. Therefore, under the same conditions, the amount of K retention via water absorption in straw is greater than that from physical adsorption and chemical adsorption. The latter two methods may, however, play an additional roles in the potassium retention.

The loss of soil nutrients (especially N and K) occurs primarily through runoff and leakage [44], [45]. In China and South Asia, rice-based cropping systems such as two- or three-cropping rotation modes are nearly universally deployed [3]. Because of the large population, food demand and soil nutrient depletion in these regions, potassium deficit has become a serious problem [46]. For these regions in particular, the combination of rain, hot and intensive farming increases the risk of nutrient losses and subsequent loss of soil fertility. The present results indicate that straw residues at different points in the decomposition process have different capacities for adsorbing potassium and retaining water. The accumulation of residues in the soil not only reduces water loss, mitigates soil erosion and provides nutrients [47], [48], but can also maintain soil nutrients, enhance soil fertility and promote the development of modern agriculture.

## Supporting Information

Figure S1 **Schematic diagram of the experimental setup of the straw decomposition trial.** The depth of the bulk soil in boxes was approximately 22 cm, and that of the flooding layer was 1 cm. The nylon bags were buried into the soil according to the orientation in the diagram.
(TIF)

Figure S2 **The adsorption of potassium (K) on wheat and rape straws for different decomposition periods.** The added K concentration is 50 mg L^−1^. The annotations in the panels are the homogeneity of variances (H) and ANOVA. The H-test was performed using the Levene test. **indicates significant differences at P<0.01. The values are the means of 3 replicates (±standard deviation). The means with the same letter are not significantly different.
(TIF)
