# Supplementary figures and images for: Dynamics of Potassium Release and Adsorption on Rice Straw Residue
Source: PLoS One. 2014 Feb 28;9(2):e90440. doi: 10.1371/journal.pone.0090440 (PMC3938734; doi:10.1371/journal.pone.0090440)

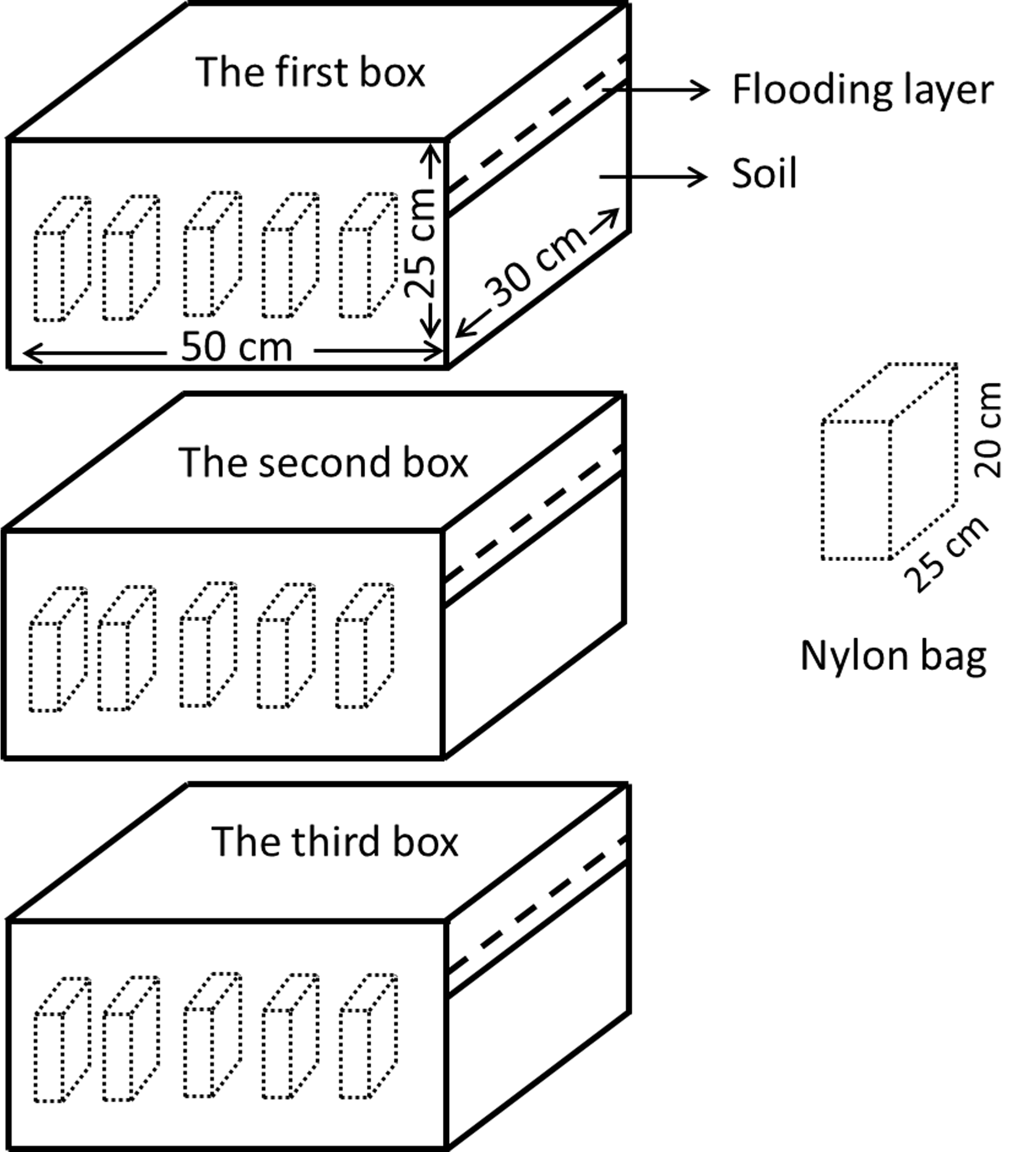

Supplement: Figure S1 — Schematic diagram of the experimental setup of the straw decomposition trial. The depth of the bulk soil in boxes was approximately 22 cm, and that of the flooding layer was 1 cm. The nylon bags were buried into the soil according to the orientation in the diagram. (TIF) [file pone.0090440.s001.tif]

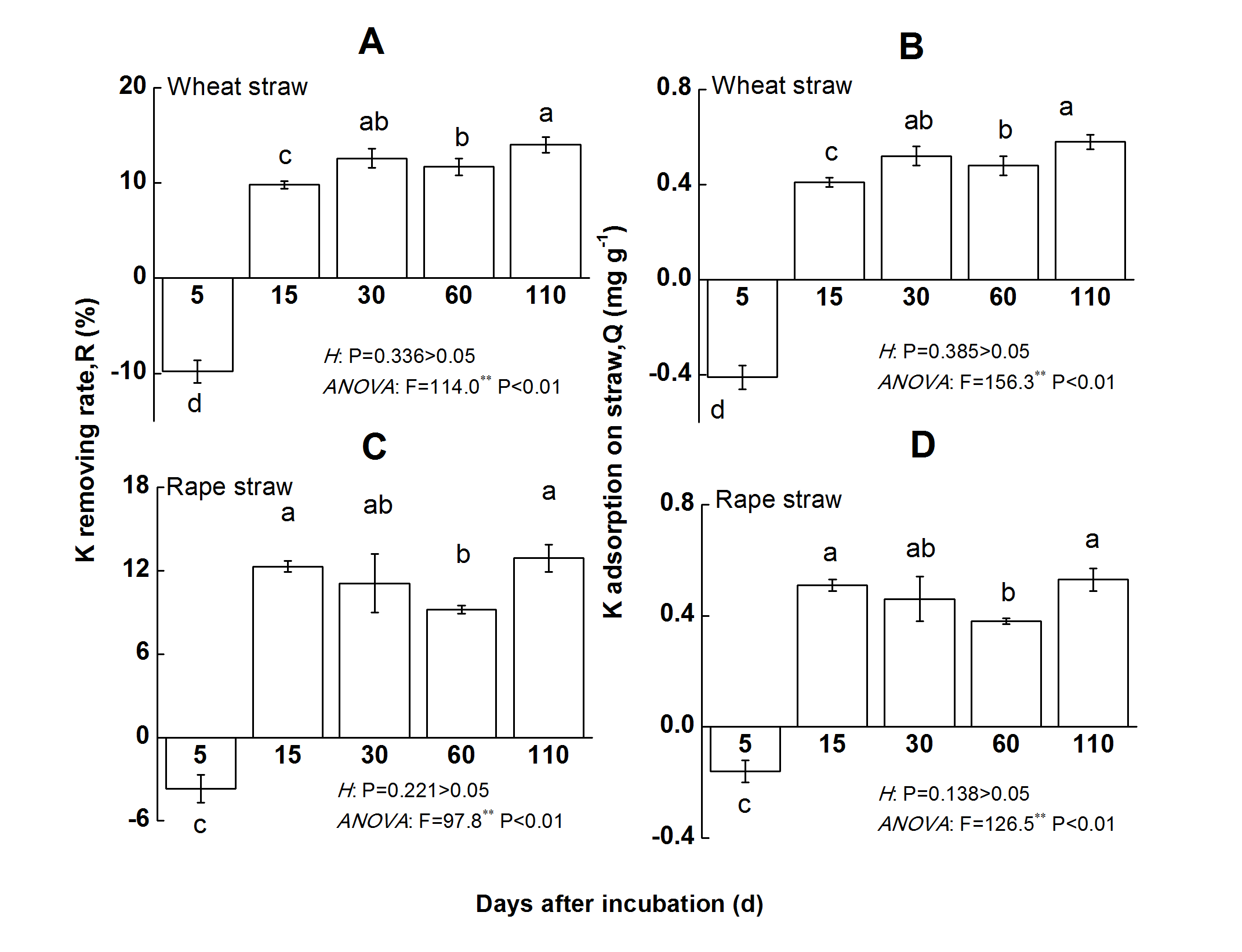

Supplement: Figure S2 — The adsorption of potassium (K) on wheat and rape straws for different decomposition periods. The added K concentration is 50 mg L−1. The annotations in the panels are the homogeneity of variances (H) and ANOVA. The H-test was performed using the Levene test. **indicates significant differences at P<0.01. The values are the means of 3 replicates (±standard deviation). The means with the same letter are not significantly different. (TIF) [file pone.0090440.s002.tif]
